# High Efficiency Water Splitting using Ultrasound Coupled to a BaTiO_3_ Nanofluid

**DOI:** 10.1002/advs.202105248

**Published:** 2022-01-27

**Authors:** Yan Zhang, Hamideh Khanbareh, Steve Dunn, Chris R Bowen, Hanyu Gong, Nguyen Phuc Hoang Duy, Pham Thi Thuy Phuong

**Affiliations:** ^1^ State Key Laboratory of Powder Metallurgy Central South University Changsha Hunan 410083 China; ^2^ Department of Mechanical Engineering University of Bath Claverton Down Bath BA2 7AY UK; ^3^ Chemical and Energy Engineering London South Bank University London SE1 0AA UK; ^4^ Institute of Chemical Technology Viet Nam Academy of Science and Technology 1A TL 29 Street, Thanh Loc Ward, District 12 Ho Chi Minh City Vietnam; ^5^ Graduate University of Science and Technology Vietnam Academy of Science and Technology 18 Hoang Quoc Viet Street, Cau Giay District Hanoi Vietnam

**Keywords:** piezoelectric, ultrasound, piezocatalysis, ferroelectric, sonochemistry

## Abstract

To date, a number of studies have reported the use of vibrations coupled to ferroelectric materials for water splitting. However, producing a stable particle suspension for high efficiency and long‐term stability remains a challenge. Here, the first report of the production of a nanofluidic BaTiO_3_ suspension containing a mixture of cubic and tetragonal phases that splits water under ultrasound is provided. The BaTiO_3_ particle size reduces from approximately 400 nm to approximately 150 nm during the application of ultrasound and the fine‐scale nature of the particulates leads to the formation of a stable nanofluid consisting of BaTiO_3_ particles suspended as a nanofluid. Long‐term testing demonstrates repeatable H_2_ evolution over 4 days with a continuous 24 h period of stable catalysis. A maximum rate of H_2_ evolution is found to be 270 mmol h^–1^ g^–1^ for a loading of 5 mg l^–1^ of BaTiO_3_ in 10% MeOH/H_2_O. This work indicates the potential of harnessing vibrations for water splitting in functional materials and is the first demonstration of exploiting a ferroelectric nanofluid for stable water splitting, which leads to the highest efficiency of piezoelectrically driven water splitting reported to date.

## Graphical abstract

By careful control of the reactor design, the formation of a stable nanofluid, and optimization of catalyst loading is demonstrated and it is possible to enhance the rate of hydrogen evolution for long‐term piezocatalysis. This has led to the highest efficiency of piezoelectrically driven water splitting reported to date.

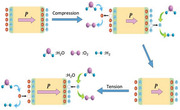

## 1 Introduction

The splitting of water to liberate hydrogen has long been promoted as a means of supplying energy in a net‐zero carbon economy.^[^ ^1^ ^]^ Improvements in technologies, such as wind and solar power, that can supply renewable energy have opened new opportunities to use the electric grid coupled with high‐performance catalytic systems to achieve hydrolysis. The use of electricity to split water can enable the production of a transportable fuel, although questions have been raised as to the full life cycle value of this approach when it may be possible to store electricity more easily in batteries.^[^ ^1^ ^]^ Photocatalysts have also been heavily investigated as a source of capturing incoming solar irradiation to split water.^[^ ^2^ ^]^ The developments in photocatalysts have been moving forward significantly and there are now complete systems with an overall cost that approaches the $1.50 threshold, as defined by the US Department of Energy (DoE) for economic viability.^[^ ^3^ ^]^ However, there remain challenges in using photocatalysts in terms of long‐term stability and overall efficiency.^[^ ^4^, ^5^, ^6^ ^]^ A new and supplementary approach to producing hydrogen from water, namely piezocatalysis, has been gaining interest in recent years and aims to exploit the spontaneous polarization of ferroelectric materials.^[^ ^7^, ^8^, ^9^, ^10^, ^11^, ^12^ ^]^

Ferroelectric materials are a class of polar materials that form an internal dipole. In the case of BaTiO_3_, which is a common ferroelectric metal oxide, the dipole is formed due to a displacement of the Ba^2+^ cation in the crystal lattice. This material exhibits a range of functionalities due to this off‐center displacement. For example, when a ferroelectric material is subjected to external pressure, an electric field is generated due to changes in the screening charges on the surface. The functional nature of a ferroelectric material has led to investigations for a range of energy conversion technologies.^[^ ^13^ ^]^ Since the 1980s there has been a recognition of the anomalous photo‐voltage for ferroelectric materials, where above band gap voltages are produced under illumination.^[^ ^14^ ^]^ This has been associated with the ferroelectric dipole and led to a range of studies to investigate the photo‐induced surface chemistry of ferroelectric materials. It has been found that photocarriers in a ferroelectric are driven by the dipole and exhibit long life times.^[^ ^15^ ^]^ Studies have also shown that thermal fluctuations can lead to changes in polarization that can be harnessed to drive chemistry, such as water splitting and disinfection. The use of mechanical vibrations,^[^ ^16^, ^17^ ^]^ induced by the application of ultrasound or mechanical stirring,^[^ ^8^, ^17^, ^18^ ^]^ is another alternative to drive a change in dipole due to stress induced changes in polarization. There have been a range of studies^[^ ^19^, ^20^ ^]^ on the displacement of a dipole for a piezoelectric material and the impact on energy harvesting. This has found applications for supplying energy for internet‐of‐things devices, structural health monitoring, and improved photovoltaic device efficiency.

The coupling of mechanical vibration to catalytic processes is a relatively new research area and has been termed piezocatalysis.^[^ ^21^, ^22^, ^23^ ^]^ This provides a fascinating new approach to water splitting and environmental remediation technologies.^[^ ^12^ ^]^ Piezocatalysis has been achieved using a variety of ferroelectric and piezoelectric materials that include lead magnesium niobate–lead titanate (PMN‐PT) single crystal,^[^ ^24^ ^]^ lead zirconate titanate (PZT),^[^ ^25^ ^]^ piezoelectric ZnO^[^ ^10^, ^11^, ^26^ ^]^ and BaTiO_3_.^[^ ^12^, ^26^, ^27^ ^]^ The process has been used to remove organic molecules, such as azo dye, from water and produce H_2_/O_2_ directly through water splitting. Due to the existence of the intrinsic internal‐built electric field in the ferroelectric, it has also been utilized as a hybrid piezo‐photo‐catalyst.^[^ ^26^, ^28^, ^29^, ^30^ ^]^ The majority of work to date has focused on morphological design, such as the formation of microfibers,^[^ ^10^, ^11^, ^25^ ^]^ nanowires,^[^ ^27^ ^]^ nanosheets,^[^ ^9^, ^28^, ^29^ ^]^ and nanoparticles.^[^ ^16^, ^31^, ^32^ ^]^ Other work has been reported on the exploitation of noble metals as the co‐catalyst^[^ ^29^, ^30^, ^33^ ^]^ and maximizing the polarization change when the working temperature is close to the Curie temperature, *T_c_*, of a ferroelectric.^[^ ^34^, ^35^ ^]^ Recent work by Su et al. indicates that multi‐phase ferroelectric materials may lead to lower energy barriers and improved catalyst performance.^[^ ^36^ ^]^

Piezocatalysis has often been examined by the application of ultrasound to ferroelectric materials within a liquid suspension.^[^ ^21^, ^23^ ^]^ There are both chemical and mechanical effects that can result from the acoustic cavitation of a liquid subjected to ultrasound.^[^ ^37^ ^]^ These effects can be powerful tools for industrial applications where chemical effects are produced during the process of free radical production and pyrolysis as a result of the local high temperature and pressure associated with cavitation events.^[^ ^38^, ^39^ ^]^ In addition, a mechanical effect is predominantly generated by shockwaves and microjets of high velocity due to cavitation events.^[^ ^37^ ^]^ The piezo‐catalytic effect, which we associate with hydrogen production in presence of a piezocatalyst, will be driven predominantly by a mechanical effect on the piezoelectric particles that changes its level of polarization due to the local stress due to cavitation, while the sono‐chemical effect (hydrogen production without a catalyst) will be driven by predominately a chemical effect. However, these two effects are rarely considered and distinguished in the study of piezocatalytic performance using driven forces induced by ultrasound; we will examine both factors in this work.

Since piezocatalysis is often carried out when the material is dispersed in water, the stability and dispersive state of the piezoelectric/ferroelectric particles within the water is also vital for long‐term catalytic performance. The effective area and dispersion of the piezoelectric material are key factors in determining performance. However, in this aspect, no exploration on the regulation and optimization of the piezoelectric particulates has been provided to date. In this work, the size, sedimentation performance, and addition of the piezoelectric will be examined in detail to provide a long‐term and stable operation for water splitting applications. A particularly novel approach, that has yet to be examined, is the formation of a stable nanofluid suspension to provide long‐term production of chemical products. Regulation of the dispersion also provides an opportunity to gain valuable insights into mechanistic designs for new improved piezocatalyic systems. This is derived from a low ionization potential and easier rotation of dipoles in the ferroelectric leading to enhanced hydrogen evolution at a rate of 270 mmol h^–1^ g^–1^.

## 2 Experimental Section

Samples of BaTiO_3_ were produced by mixing the appropriate amounts of BaCO_3_ and TiO_2_ powders and high‐speed ball milling for 24 h. These materials were then calcined at 800 °C for 3 h before cooling to room temperature. The resulting powder was ball milled for 24 h before applying a second heat treatment to 1200 °C for 1 h to form the ferroelectric tetragonal phase of the material. X‐ray diffraction (XRD) patterns of the powders were obtained with a Bruker D8 advance diffractometer using Cu‐K*α* radiation. High‐resolution scans were obtained in a continuous scan mode at a scan speed of 0.6 ° min^−1^ with a collection width of 0.0167°. The morphology of the powders was observed using a scanning electron microscope (SEM, FEI Inspect F) and transmission electron microscope (TEM, JEOL JEM 2010). The mean particle size was measured by analyzing the SEM micrographs and the BET surface area of the powders was obtained on the Micromeritics Gemini VII surface area analyzer using N_2_ as the adsorptive gas. The lattice phase of the initial (fresh) and spent catalysts were studied by Raman spectroscopy on an XploRATM Plus (Horiba, Japan) with laser excitation at 535 nm. To avoid the phase transition which may be induced by thermal treatment, after being collected from the reaction solution by sedimentation, the spent powders were dried naturally at room temperature prior to Raman analysis. The elemental composition of the prepared BTO was confirmed by energy dispersive X‐ray spectroscopy (EDS) analysis on a JEOL IT‐ 200.

**Figure 1:**
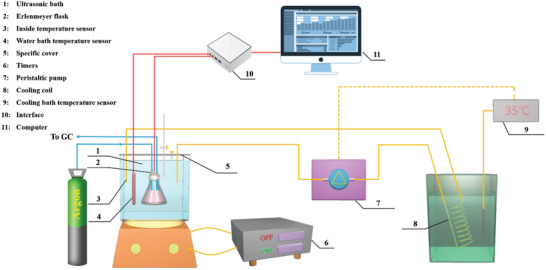
Experimental arrangement of in‐line gas analysis. 1. Ultrasonic bath, 2. Erlenmeyer flask, 3. Solution temperature sensor, 4. Water bath temperature sensor, 5. Timers, 6. Peristaltic pump, 7. Cooling coil, 8. Cooling bath temperature sensor, 9. Interface, 10. Computer and 11. Flexible stand.

A schematic of the experimental arrangement is shown in **Figure** **1**. Sono‐ and piezo‐catalysis of the samples was performed in a 150 ml Erlenmeyer flask containing 100 ml of 10% methanol solution in which methanol was used as a sacrificial reagent to scavenge OH radicals.^[^ ^40^, ^41^ ^]^ A measured amount of the BaTiO_3_ was added to the flask; in addition, non‐ferroelectric particles were also studied as a control material, namely TiO_2_. The solution was then stirred on a magnetic stirrer for times ranging from 15 to 60 min. The flasks were then sealed and de‐gassed with Ar for 45 min prior to being located in the center of a 60 W ultra‐sonic bath (60W‐TP01‐Taiwan Total Meter). The process of both sono‐ and piezo‐catalysis relies upon cavitation events, thus, they are strongly influenced by the geometry and height of the water inside the bath.^[^ ^42^ ^]^ Therefore, to eliminate bias of comparison as a result of unavoidable and uncontrollable evaporation of water inside the ultrasonic bath, sono‐ and piezo‐catalysis were conducted for 90 min unless stated otherwise. A specifically designed cover was used to maintain the Erlenmeyer flask at a desired position which was 10 mm or 13 mm with respect to the base of the ultrasonic bath; these two distances were selected to vary the acoustic intensity and study how this affects the efficiency of piezocatalysis. The frequency of sonication was 40 kHz and a constant bath temperature of 35 °C ± 1 °C was maintained by circulating water through an external cooling bath during the experiment. The particle size after sonication was measured by dynamic light scattering (DLS) using a Nanoparticle Analyzer (Horiba SZ‐100).

The acoustic power (*P_A_*), which is expressed in *W*, absorbed by a known amount of water in the Erlenmeyer flasks was estimated by calorimetric measurements ^[^ ^43^, ^44^ ^]^ and calculated by Equation (1):

(1) $$P_{A}=C_{P}\;m\frac{dT}{dt}$$

where *C_P_*, *m* and d*T*/d*t* are the heat capacity of water at constant pressure (*C_P_* = 4.178 J g^–1^ K^–1^), the amount of water (g), and the ramp rate of temperature (K s^–1^), respectively. Figure S1, Supporting Information shows the temperature profiles of calorimetric measurements which were used to determine the thermal ramp rate when the reactor vial was filled with different volumes. Consequently, the acoustic intensity (which is expressed in W L^–1^) was calculated as the acoustic power (*P_A_*) divided by the volume (*V*) of the irradiated liquid. All calorimetric measurements were performed in triplicate.

For online measurements of the product formed during catalysis, the generated hydrogen was continuously swept by an argon stream at approximately 12 ml min^–1^ and passed to a 20 ml‐condensation vial before being sent to a gas chromatograph (GC), Agilent model HP 5890 Series II, that was equipped with a thermal conductivity detector and RT‐Msieve 13X capillary column (30 m × 0.32 mm, Thames Restek) by ChemStation Software. Actual flow rates were determined with a soap‐film bubble flowmeter (Hewlett‐Packard) during each run. The hydrogen production rate was calculated using a calibration curve that was produced using different concentrations of hydrogen diluted in argon according to Equation (2) and Equation (3).

(2) $$F_{H_{2}}^{i}=C_{H_{2}}^{i}\;\;\times F_{\mathrm{total}}^{i}$$

(3) $$Q_{H_{2}}^{i}=\frac{{\left[F_{H_{2}}^{i}\right]}_{\mathrm{cat}}-{\left[F_{H_{2}}^{i}\right]}_{\mathrm{blank}}}{m_{BTO}}\;$$

where $F_{H_{2}}^{i}$ is the instantaneous molar flow rate of hydrogen (µmol h^–1^)

$C_{H_{2}}^{i}$ is the instantaneous hydrogen concentration determined by GC every 3 min of sonication (µmol ml^–1^)

$F_{\mathrm{total}}^{i}$ is the actual flow rate determined before each GC analysis (ml h^–1^)

$Q_{H_{2}}^{i}$ is the instantaneous piezo‐catalytic production rate of hydrogen (mmol h^–1^ g^–1^)

${\left[F_{H_{2}}^{i}\right]}_{\mathrm{cat}}$ is the instantaneous molar flow rate of hydrogen in the presence of a catalyst (µmol h^–1^)

${\left[F_{H_{2}}^{a}\right]}_{\mathrm{blank}}$ is the average molar flow rate of hydrogen without a catalyst at steady state (µmol h^–1^)

*m_BTO_* is the mass of BaTiO_3_ added to the solution (mg)

## 3 Results and Discussion

**Figure 2:**
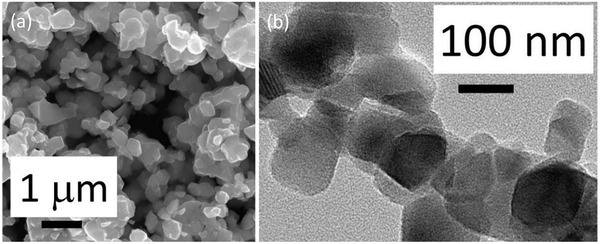
SEM a), and TEM b) micrographs of typical BaTiO_3_ sample after second thermal cycle.

A typical sample of the BaTiO_3_ powders was investigated by electron microscopy, as shown in **Figure** **2**. Figure 2a shows an SEM micrograph that indicates there are agglomerated particles of approximately 400 nm in size. The panel in Figure 2b shows a TEM micrograph of typical particles and enables observation of the finer detail of the primary particles that are producing the larger agglomerated particles shown in the SEM. The TEM image indicates that the agglomerates in the SEM micrograph are made up of smaller primary particles approximately 100  to 150 nm in size. A more detailed investigation shows that the average size of 50 particles imaged from SEM was determined as 398 nm, with a BET surface area of 2.048 m^2^ g^−1^. The particle size and surface area of a catalyst significantly impact the overall efficiency of a catalytic system.

**Figure 3:**
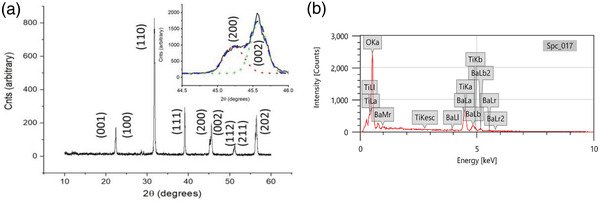
a) Wide angle diffraction patterns for BaTiO_3_ and (inset) small angle investigation around 45 2*θ* showing peak splitting for (002)(200) indicating tetragonal symmetry. b) EDS result of the as‐prepared BaTiO_3_

The phase of the BaTiO_3_ powder was analyzed using X‐ray diffraction with the wide‐angle 2*θ* of 10–60°, whose diffraction pattern is shown in **Figure** **3** (a). There is good agreement with the expected diffraction patterns for BaTiO_3_ since pure cubic BaTiO_3_ (*c*‐BaTiO_3_) exhibits a single peak at 2*θ* = 45° (PDF 31 0174) and this peak is assigned to the (200) lattice plane. For tetragonal BaTiO_3_ (*t*‐BaTiO_3_) the peak at 2*θ* = 45° shows a split at 2*θ* = 44.8° for (002) and 2*θ* = 45.4° for (200) (PDF 05 0626). This peak splitting can be used to determine whether the BaTiO_3_ is cubic (non‐ferroelectric) or tetragonal with the possibility of ferroelectricity associated with the tetragonal phase. The small angle patterns for BaTiO_3_ appear to exhibit a single peak around 2*θ* = 45°. This peak has a shoulder that is indicative of splitting with two lattice structures superimposed.

A detailed investigation of the high‐resolution diffraction pattern of the region around 2*θ* = 45° can be seen in the inset of Figure 3a. The peaks obtained are broad, indicating a small primary crystallite size for the material. Application of the Scherrer formula provides a lower limit in the range of 50 nm for the crystallites. The X‐ray data shows clear peak splitting indicative of a tetragonal crystal lattice. Therefore, the grains seen in Figure 2 are likely to be formed from smaller crystallites of the two crystallographic phases of BaTiO_3_, namely cubic (non‐ferroelectric, paraelectric) and tetragonal (ferroelectric).

The elemental composition of the BaTiO_3_ powder was determined by EDS analysis, and is shown in Figure 3b. It was confirmed that the as‐prepared powder is exclusively composed of Ba, Ti, and O elements with their atomic percentage of 22%, 21%, and 57%, respectively, that relates to BaTiO_3._

During the water splitting experiments, that were performed in 10% MeOH solution, a number of visual observations were made that led to further detailed analysis of the process. The most interesting observation was that when the BaTiO_3_ powder was shaken or stirred prior to the application of ultrasonic agitation a cloudy suspension was formed. Ultrasonic agitation leads to the production of bubbles through cavitation that form and subsequently collapse to produce areas of high pressure in the order of 1 GPa.^[^ ^45^ ^]^ The standing waves produced by the applied ultrasound can also lead to areas where there is limited agitation and can lead to a build‐up of material in these regions. These high‐pressure events couple with the piezoelectric material to produce deformation and produce local electric fields that drive surface dissociation of chemisorbed species. If there was no initial stirring/shaking prior to the application of ultrasound a large number of agglomerated BaTiO_3_ particles remained in the base of the flask, as can be seen in Figure S2, Supporting Information, and these agglomerated particles did not contribute to the evolution of hydrogen.

Measurements of the particle size after 5, 20, and 90 minutes of ultrasound show that there is a reduction in average size with ultrasound time. In the case of pre‐stirred solutions, the average particle size reduces to approximately 150 nm after 90 minutes of ultrasound. If there is no stirring/shaking prior to the application of ultrasound the average particle after 90 min of ultrasound is larger at 180 nm. Details of the change in particle size during ultrasonic agitation can be found in Figure S3, Supporting Information.

**Figure 4:**
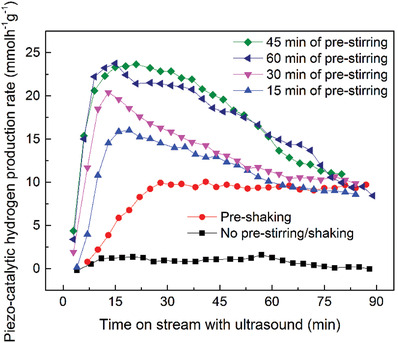
Hydrogen evolution for a BaTiO_3_ load of 100 mg l^–1^ in 10% MeOH water solution volume at 10 mm location above bottom of ultrasonic bath. A trend towards a hydrogen evolution rate of 10 mmol h^–1^ g^–1^ can be seen.

The effect of agitating the sample by stirring/shaking prior to the application of ultrasound had a noticeable impact on hydrogen generation, as can be seen in **Figure** **4**. When the sample had no agitation prior to the application of ultrasound there was minimal hydrogen evolved, whereas when the sample had been agitated prior to the application of ultrasound there was measurable hydrogen produced. The hydrogen evolution rates presented in Figure 4 are therefore of interest in that they demonstrate a higher rate of evolution when the ultrasound is initially applied.

The evolution rate then normalizes over time, where all pre‐stirred and pre‐shaken tests converge to a similar hydrogen production rate. In a typical catalytic environment, it might be expected that the rate of hydrogen evolution would initially be slow and then increase as the catalyst undergoes surface reconstruction and pre‐conditioning under the external energy source. However, we believe that ferroelectric materials behave in an anomalous manner as a result of their inherent dipole, since this can lead to a strong Inner Helmholtz Layer (IHL), where the IHL can result in polar species being tightly bound to the ferroelectric. A combination of the production of a fine scale suspension due to reduction in BaTiO_3_ particle size (Figure S3), and the release of species held in the IHL can explain the high initial rate of hydrogen evolution that then normalizes to a similar rate for all systems. This hypothesis is further supported by the results obtained for pre‐shaking as opposed to pre‐stirring. In the case of pre‐shaking the flask was vigorously agitated by hand, which was a more aggressive process, thereby providing enough energy to disturb the IHL so that system undergoes some pre‐conditioning with an increase in hydrogen evolution until steady‐state is reached.

We further investigated the effects of changing the loading level of BaTiO_3_ in a range of 5 to 100 mg l^–1^ and the location of the flask with respect to the base of the ultrasonic bath, 10  and 13 mm from the base, on hydrogen evolution. The acoustic power contained inside the reactor varies with location in the ultrasonic bath due to reflection and resonance effects of the ultrasound. The high reproducibility was ensured by our experimental setup, which can be seen in Figure S4, Supporting Information.

**Figure 5:**
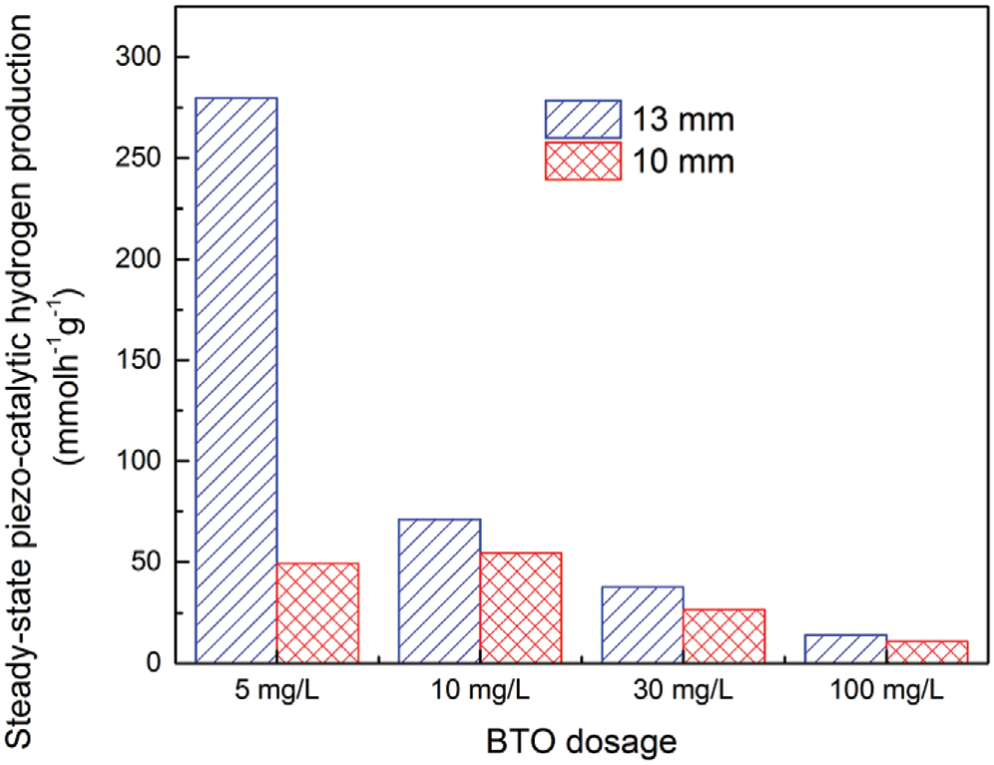
Steady‐state (90 min of ultrasound) piezo‐catalytic hydrogen production at position 10 mm and 13 mm above bottom of ultrasonic bath in 10% MeOH water solution. An influence of BaTiO_3_ loading and position relative to the ultrasonic bath can be observed.

It was found that there is an optimum distance, *h*, from the transducer in a vertical direction, namely from the bottom of the ultrasonic bath, for the reactor to be placed to obtain the highest intensity of ultrasound.^[^ ^39^, ^43^ ^]^ Our results also showed that the acoustic intensity in the Erlenmeyer flasks placed at a 13 mm height from the base was 1.3 times higher than at 10 mm from the base (34 and 26 W L^–1^, respectively). In an ultrasonic water bath, ultrasound is transmitted from the transducer, which is usually located under the base of the bath, to the water in the bath, and finally to the reaction solution in the Erlenmeyer flask. It is also reflected both from the tank and flask walls and from the water surface, resulting in the generation of geometry‐dependent standing waves. As a result, different regions of energy density are located within the bath. Marangopoulos et al.^[^ ^42^ ^]^ experimentally showed that it is possible to observe differences of a factor of ten between the maximum and minimum energy densities at different *z*‐positions even at the same *x*,*y*‐axis position. Increasing the acoustic intensity induces an increase in both chemical and mechanical effects, which are mainly due to increasing bubble collapse temperature, bubble collapse time, and number of bubbles.^[^ ^37^, ^38^, ^46^ ^]^ Consequently, a larger acoustic intensity at the 13 mm location resulted in both higher piezocatalytic activity and higher collision probability/agglomeration compared to those at the 10 mm location. In addition, a higher loading dose of BaTiO_3_ also results in both higher collision probability/agglomeration and a higher shielding effect, in which particles will absorb vibration energy and prevent ultrasound from penetrating further into the reaction vial, that cause a decrease in hydrogen production. Steady‐state hydrogen evolution rates for 10  and 13 mm heights at various BaTiO_3_ loads are presented in **Figure** **5**. It can be seen that after 90 minutes of ultrasound there is a higher level of hydrogen evolution per gram at lower loading doses. At a position of 10 mm, the maximum rate of evolution was 53 mmol h^–1^ g^–1^ for a loading level 10 mg l^–1^, and then decreased to 11 mmol h^–1^ g^–1^ at a higher loading level of 100 mg l^–1^. When the flask position was raised to 13 mm from the base of the ultrasonic bath, an increase in hydrogen evolution was observed for all loading doses compared to the 10 mm location. However, an exceptional increase in hydrogen evolution per gram was found for a low loading dose of 5 mg l^–1^, which is much higher than that observed for the higher loading dose. This confirms that increasing the acoustic intensity results in an enhanced piezo‐catalytic hydrogen evolution, especially at low loading dose levels. Therefore, the threshold dosage, at which almost no collision probability/agglomeration and shielding effects occur, should be carefully considered case by case to maximize the piezo‐catalytic activity of any ferroelectric materials.

**Figure 6:**
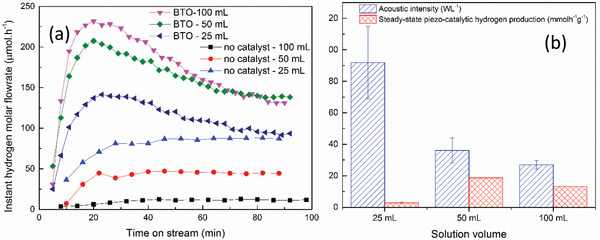
Molar flowrate of hydrogen evolution a) and steady‐state (determined at 90 min of ultrasound) hydrogen evolution b) obtained using different solution volumes at 10 mm location above bottom of the ultrasonic bath. BaTiO_3_ load of 100 mg l^–1^ in 10% MeOH water solution.

While the reproducibility of the experiment at BTO dosage of as low as 5 mg l^–1^ was confirmed (see Figure S4, Supporting Information), weighing such a small amount is challenging. Therefore, a BTO dosage of 100 mg l^–1^ was used for further experiments to investigate the effect of changing the acoustic intensity by changing the solution volume inside the Elermeyer flask, while maintaining the same BTO loading dose. **Figure** **6a** clearly shows that there is a trend for a higher rate of sono‐chemical hydrogen evolution at a higher acoustic intensity, while there is an optimum acoustic intensity that results in the highest piezocatalytic hydrogen production. As mentioned, sono‐chemical hydrogen evolution is driven by chemical effects that are enhanced at a higher acoustic intensity. In addition, an increase in acoustic intensity may result in both an increase in piezocatalytic activity due to a higher mechanical effect and an increase in collision probability/agglomeration and shield effecting due to a higher number of generated cavitation bubbles. While an increase in the number of collisions leads to an increase in piezocatalytic hydrogen evolution, shielding can limit it, resulting in an optimum acoustic intensity for the highest piezocatalytic hydrogen production, as can be seen in Figure 6b.

Using the higher heating value (HHV) for hydrogen of 286 kJ mol^–1^ and the hydrogen evolution of 132 µmol h^–1^, which was obtained for 10 mg l^–1^ loading, for a 100 ml solution at a 10 mm location we calculate this to be ≈0.011 W of hydrogen.^[^ ^47^ ^]^ With the acoustic power of 2.7 W, which was transferred to the solution inside the Erlenmeyer flask, an overall efficiency of approximately 0.41% for the generation of hydrogen, which is defined as a ratio between the hydrogen produced and electricity consumed, was obtained.

**Figure 7:**
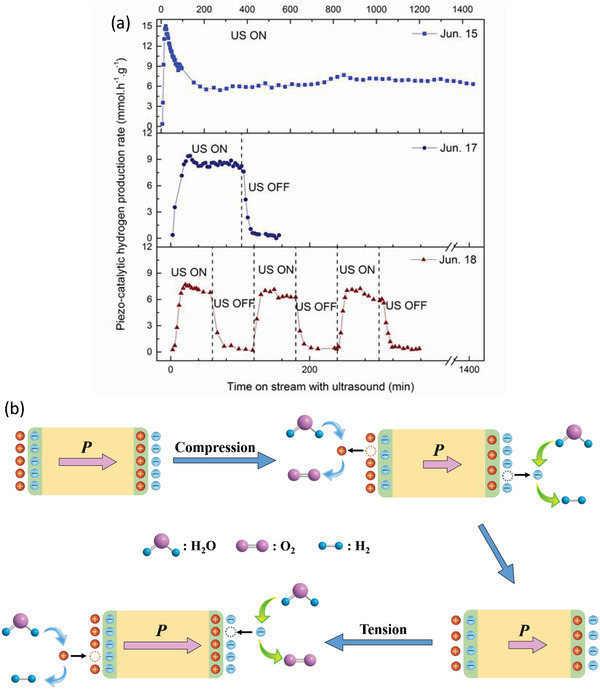
a) Stability tests for BaTiO_3_ (100 mg l^–1^) in 10% MeOH solution at 10 mm. b) Schematic of the charge development on the surface of the ferroelectric particle under the alternating compression and tension forces realized by the application of ultrasound for water splitting.

A key consideration in any catalytic system is the stability of the catalyst. In order to determine whether the BaTiO_3_ was behaving as a catalyst or sacrificially participating in the reaction, a single 24 h run, that was then followed by sequential 90 min tests, were performed, as summarized in **Figure** **7a**. The 24 h run, seen in the upper panel of Figure 7a, shows that once steady state hydrogen evolution is reached this is stable for the duration of the whole 24h test. The middle and bottom panels of Figure 7 shows the influence of leaving the system for a period before switching off and restarting the ultrasound. There is a slight increase in hydrogen evolution at the beginning of the ultrasound that declines as steady state evolution is reached; this is likely to be related to the generation of tightly bound species on the interfaces between BaTiO_3_ and the solution due to a need for screening. Figure 7b illustrates the mechanism of H_2_ and O_2_ generation produced by BaTiO_3_ powders for piezo‐catalysis. The spontaneous polarization of the ferroelectric ceramic is assumed to be the main driving force during the piezoelectric catalysis process. During the application of ultrasound, it propagates through the fluid by a series of compression and rarefaction (expansion) waves which exert the compression and tension force to the particle in the solution. Sufficient piezoelectric charges with opposite signs (*q^+^* and *q^–^*, Equation 4) can be generated by the discontinuous compressive and expansion stress under ultrasonic oscillation. The water molecules are oxidized by *q^+^*, shown in Equation 5, leading to the formation of H^+^ and O_2_. The correspondingly produced *q^–^* can be used for the H_2_ generation in Equation 6. Therefore, the changed piezoelectric potential formed by the application of ultrasound drove the electron transfer between the ferroelectric surface and involved ions such as H^+^/OH^–^ for H_2_ generation:

(4) $$\begin{array}{c} \left(\sigma \right)\\ \mathrm{BaTi}O_{3}\to \mathrm{BaTi}O_{3}+q^{-}+q^{+} \end{array}$$

(5) $$H_{2}O+2q^{+}\to 2H^{+}+\frac{1}{2}O_{2}$$

(6) $$2H^{+}+2q^{-}\to H_{2}$$

**Figure 8:**
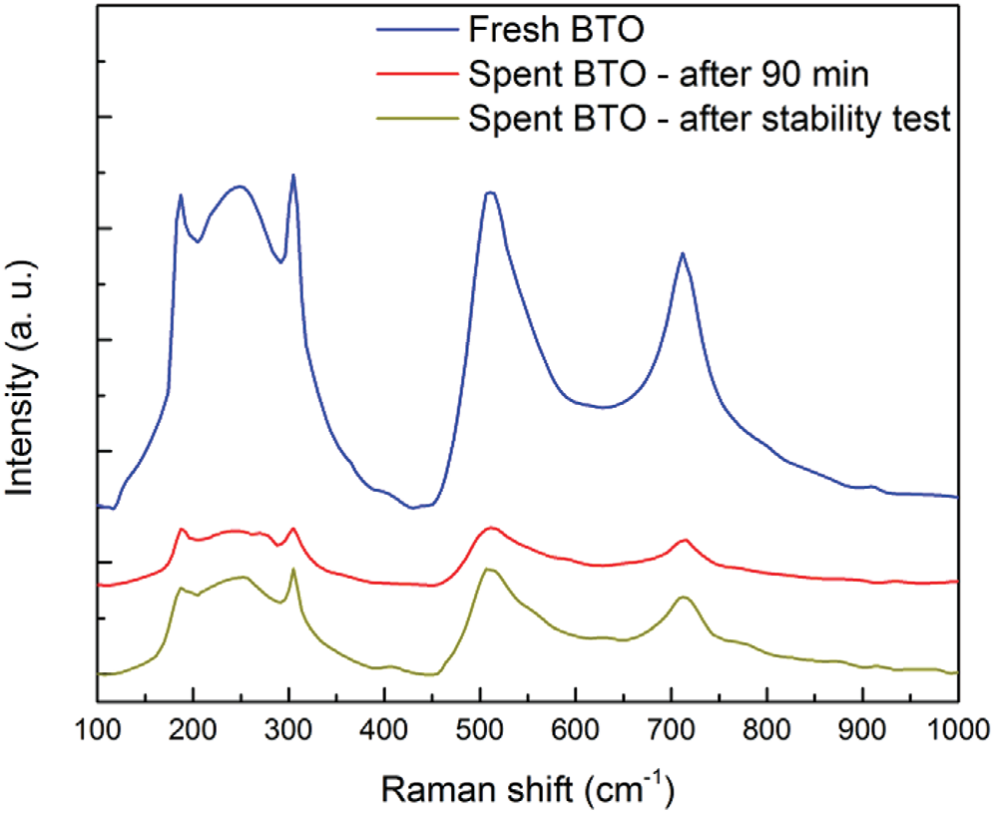
Raman spectra of the fresh and spent BTO after 90 min and stability test (BTO dosage of 100 mg·L^–1^ in 10% MeOH solution at 10 mm).

Determining the level of tetragonality from XRD spectra may cause some uncertainty due to the peak broadening effect. Therefore, Raman spectroscopy has been widely used to study the tetragonal‐cubic symmetry of BTO.^[^ ^48^, ^49^, ^50^ ^]^ It is known that the sharp peak at ≈305 cm^–1^ indicates the dominant tetragonal phase.^[^ ^48^, ^50^ ^]^ Lee et al. ^[^ ^49^ ^]^ observed that its relative intensity gradually decreased with a decrease in the degree of tetragonality of BTO. As can be seen in **Figure** **8**, the scattering intensities declined significantly in the Raman spectra for the spent catalyst compared to the initial (fresh) catalyst, confirming the particle size reduction due to cavitation events produced by the ultrasound. However, the relative intensity of the peak at 305 cm^–1^ remained unchanged, indicating that the BTO particles are not changing phase during ultrasound, thereby providing potential for long‐term operation if the particles are suspended in a stable nanofluid.

**Figure 9:**
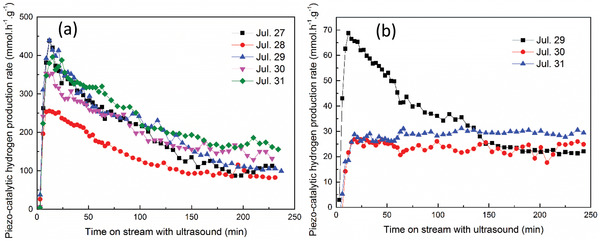
Stability tests with a BTO dosage of a) 5 mg l^–1^, and (b) 30 mg l^–1^ (pre‐stirring: 45 min, solution: 10% MeOH, distance h: 13 mm).

The formation of a stable nanofluid suspension is a critical condition to ensure the efficiency and stability of a piezocatalyst. Pre‐stirring/shaking followed by ultrasonication is able to generate a stable BaTiO_3_ nanofluid in which its particle size reduces from an initial distribution of approximately 400 nm as fabricated (see Figure 2) to a distribution of approximately 150 nm after 90 min of applying ultrasound at all catalyst loading dose (see Figure S5, Supporting Information). Sadeghi et al.^[^ ^51^ ^]^ stated that a suspension will be mono‐disperse if the polydispersity index (PDI) which can be determined by dynamic light scattering (DLS) analysis is lower than 0.3. Thus, it can be seen that a mono‐disperse state was quickly reached after 5 min of ultrasonication and remained stable during the first 90 min, as shown in **Table** **1**. However, after 240 min of ultrasonication, a mono‐disperse state was only maintained for a loading of 5 mg l^–1^ of BaTiO_3_, while it was broken for higher loading doses. A much higher particle size was also observed at higher catalyst loading dose by both DLS analysis (see Figure S5, Supporting Information and Table 1) and by visual inspection, see Figure S6, Supporting Information. While it is known that ultrasonication can break down particle clusters, agglomeration of aggregation of the nanoparticles can result from prolonged ultrasonication.^[^ ^52^, ^53^, ^54^ ^]^ Deagglomeration can occur again by applying ultrasound, while aggregation is irreversible ^[^ ^55^ ^]^ so that aggregated particles cannot be broken down. Since irreversible aggregation is enhanced by particle crowding, a higher catalyst loading dose exhibited a higher possibility of aggregation, and this results in a lower, but stable, hydrogen production rate for the subsequent working days as can be seen in the case of using the loading doses of 30 mg l^–1^ at **Figure** **9b** and 100 mg l^–1^ of BaTiO_3_ at Figure 7. At a much lower loading dose of 5 mg l^–1^, a similar trend in hydrogen production rate with time on stream was observed for the first and next four working days as can be seen in Figure 9a. Together with the DLS and sedimentation results, this could be a sign of a stable nanofluid with no irreversible aggregation occurred during ultrasonication, making the loading dose of 5 mg l^–1^ of BaTiO_3_ the optimum that exhibited the highest piezocatalytic activity and stability.

**Table 1:** The Z‐average diameter (Z‐aver) and polydispersity index (PdI) were obtained from DLS

Dose [mg l^–1^]	Z‐aver [nm]				PdI			
	5 min	20 min	90 min	240 min	5 min	20 min	90 min	240 min
100	185	172	156.4	1091.5	0.237	0.091	0.181	0.525
30	181.4	165.7	152.8	485.9	0.153	0.049	0.113	0.321
5	185.2	169.1	151.1	146.1	0.142	0.063	0.028	0.105

**Figure 10:**
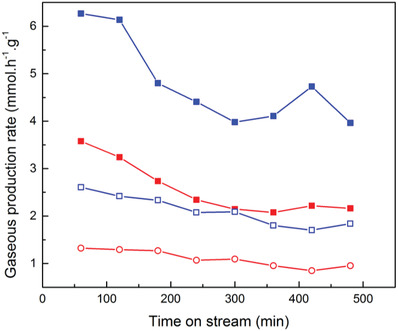
Hydrogen (square symbols) and oxygen (circle symbols) evolution demonstrate a 2:1 ratio as expected for water splitting at 10 mg l^–1^ (solid symbols) and 100 mg l^–1^ (open symbols).

In order to demonstrate that the process is related to splitting water a measurement of the hydrogen and oxygen evolution in the absence of a scavenger was performed. The results shown in **Figure** **10** indicate that the expected 2:1 ratio of hydrogen to oxygen is being produced by the catalytic reaction, and the higher loading dose (open symbols) results in lower piezocatalytic activity.

To test whether the hydrogen evolution was due to sonocatalysis, rather than piezocatalysis, a non‐ferroelectric control (Figure S7, Supporting Information) sample was tested based on nanostructured TiO_2_ and we observed negligible hydrogen evolution. The rate of evolution of hydrogen found in our system can be explained through the mixture of cubic and tetragonal BaTiO_3_ and the production of suspended nanoparticles. Su et al. previously demonstrated that when two phases of BaTiO_3_ are present there is a low energy barrier to rotation of polarization. The observation of this effect has been observed previously in doped systems. Here, and in the work of Su et al., such an effect seems to be present in the nanostructured BaTiO_3_ due to the high surface area of interfaces and relaxation of surface free energy that drives the chemical reaction. This leads to a range of phases being present and an enhanced electro‐mechanical coupling due to the ability of external stress to cause crystallographic flexibility. These changes come together to produce a system that is capable of converting the mechanical energy of ultrasound into catalysis through the piezoelectric effect.

**Figure 11:**
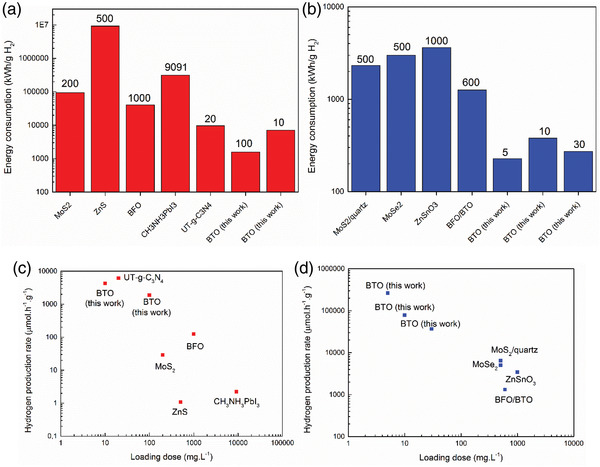
Comparison of the performance of different piezocatalysts toward water splitting in the absence a,c) and presence of methanol b,d) from our work and others in terms of energy efficiency‐kWh g^–1^ of produced hydrogen (a & b) and hydrogen production rate‐µmol h^–1^ g^–1^ (c & d). Number in graphs (a & b) indicated catalyst loading dose (mg l^–1^). Energy consumption is calculated based on the power of the ultrasonic bath (W) and hydrogen production rate (g h^–1^).

**Figure** **11** provides a summary of the efficiencies of using different piezocatalysts at different loading doses both in terms of hydrogen evolution rate and energy consumption, which have been reported in the literature compared with those obtained from this work. It is clear that by optimization of the catalyst loading dose and the formation of a stable suspension, a promising piezo‐catalytic hydrogen production rate of 4.5 mmol g^–1^ h^–1^ is achieved from pure water, which is comparable to an exceptional piezo‐catalytic hydrogen production rate that was reported from graphitic carbon nitride nanosheets (6.2 mmol g^–1^ h^–1^)^[^ ^16^ ^]^ and is a much larger rate than obtained from other piezocatalysts such as BiFeO_3_ nanosheets (BFO),^[^ ^9^ ^]^ ZnS nanosheets,^[^ ^56^ ^]^ MoS_2_,^[^ ^57^ ^]^ and an organo‐lead halide perovskite CH_3_NH_3_PbI_3_.^[^ ^58^ ^]^ The addition of a small amount of methanol clearly improves the piezocatalytic efficiency both in terms of hydrogen production rate and energy consumption. The maximum rate of H_2_ evolution found was 270 mmol h^–1^ g^–1^ and lowest energy consumption of 227 kWh g^–1^ H_2_ (calculated based on the power of the ultrasonic bath) or 10 kWh g^–1^ H_2_ (calculated based on the transferred acoustic power) for a loading of 5mg l^–1^ of BaTiO_3_ in 10% MeOH/H_2_O leading to the highest efficiency of piezoelectrically driven water splitting that has been reported to date.^[^ ^40^, ^59^, ^60^ ^]^

## 4 Conclusions

We have provided the first demonstration that the production of a stable nanofluid suspension of ferroelectric BaTiO_3_ nanoparticles composed of a mixture of cubic and tetragonal crystallites can lead to long‐term piezocatalytic water splitting under the application of ultrasound. The primary particles break up into smaller particles during the first 90 minutes of ultrasound and then move to a steady‐state of water splitting into hydrogen and oxygen. By varying the loading level of BaTiO_3_ to produce a stable nanofluid we maximize the hydrogen evolution to approximately 250 mmol h^–1^ g^–1^. Long‐term evaluation of performance shows that the system is stable and produces the predicted 2:1 ratio of hydrogen and oxygen for water splitting. The insights from this work also show that careful reactor design, formation of a stable nanofluid, and optimum catalyst loading can be used to enhance the rate of hydrogen evolution and piezocatalysis. This has led to the highest efficiency of piezoelectrically driven water splitting reported to date.

## Conflict of Interest

The authors declare no conflict of interest.

## Supporting information

Supporting Information

## Data Availability

The data that support the findings of this study are available from the corresponding author upon reasonable request.
