# Supplementary material for: High Efficiency Water Splitting using Ultrasound Coupled to a BaTiO3 Nanofluid
Source: Adv Sci (Weinh). 2022 Jan 27;9(9):2105248. doi: 10.1002/advs.202105248 (PMC8948565; doi:10.1002/advs.202105248)
Supplement: Supplementary file 1 — Supporting Information [file ADVS-9-2105248-s001.pdf]

ADVANCED SCIENCE
Open Access

Supporting Information

for *Adv. Sci.,* DOI: 10.1002/advs.202105248

High efficiency water splitting using ultrasound coupled to a BaTiO3 nanofluid

Yan Zhang‡, Hamideh Khanbareh, Steve Dunn", Chris R Bowen, Hanyu Gong, Nguyen Phuc Hoang Duy‡, Pham Thi Thuy Phuong**

High efficiency water splitting using ultrasound coupled to a $BaTiO_3$ nanofluid

Yan Zhang[1‡], Hamideh Khanbareh[2], Steve Dunn[3″], Chris R Bowen[2*], Hanyu Gong[1], Nguyen Phuc Hoang Duy[4‡], Pham Thi Thuy Phuong[4, 5*]

1 State Key Laboratory of Powder Metallurgy, Central South University, Changsha, 410083, Hunan, China

2 Department of Mechanical Engineering, University of Bath, Claverton Down, Bath, BA2 7AY, UK

3 Chemical and Energy Engineering, London South Bank University, London, SE1 0AA

4 Institute of Chemical Technology, Viet Nam Academy of Science and Technology, 1A TL 29 Street, Thanh Loc Ward, District 12, Ho Chi Minh City, Vietnam

5 Graduate University of Science and Technology, Vietnam Academy of Science and Technology, 18 Hoang Quoc Viet Street, Cau Giay District, Hanoi, Vietnam

‡ Those authors contributed equally

* Corresponding authors: pttphuong@ict.vast.vn; dunns4@lsbu.ac.uk; c.r.bowen@bath.ac.uk

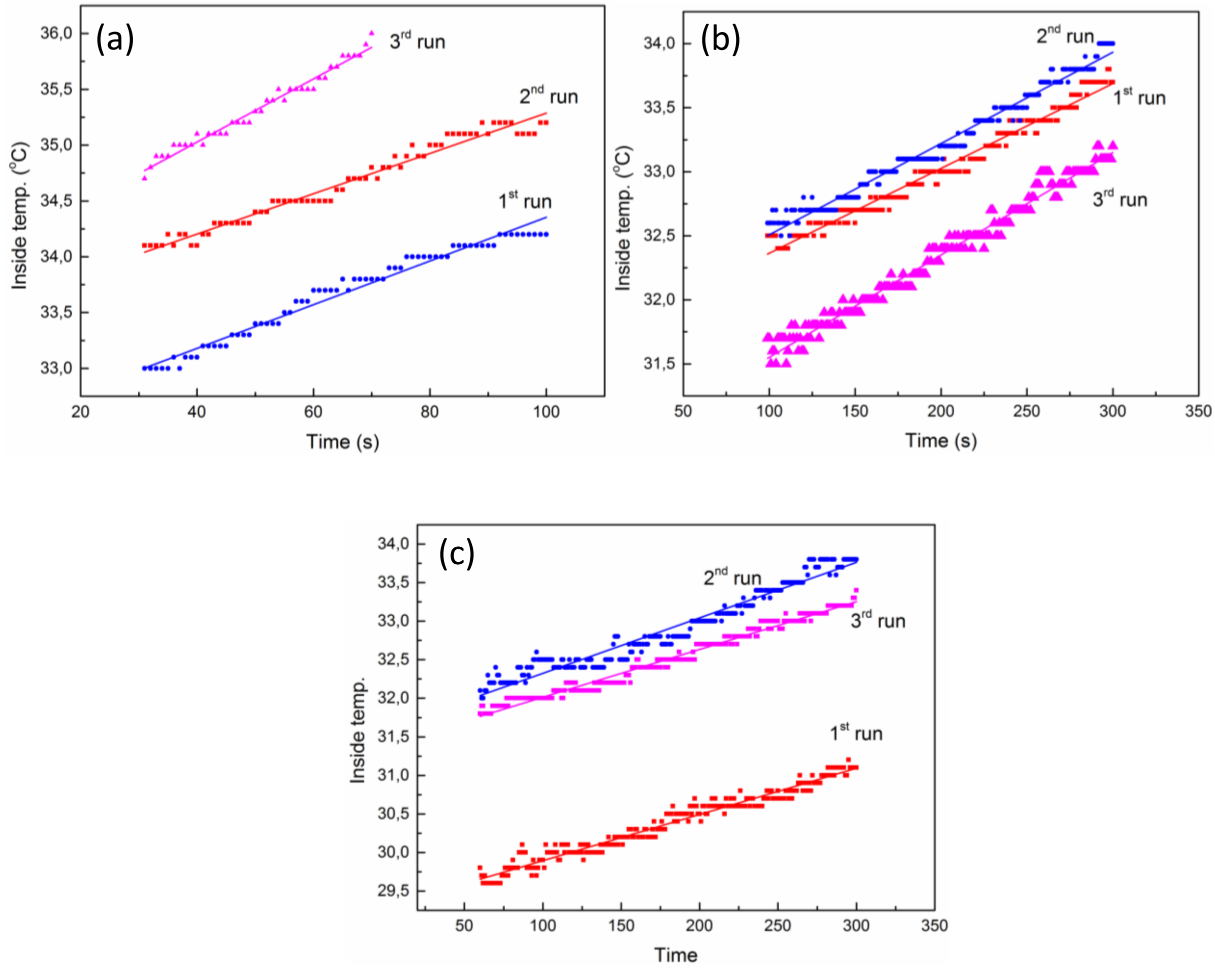

Figure S1. Temperature-time profiles of calorimetric measurements when using (a) 25 mL, (b) 50 mL, and (c) 100 mL.

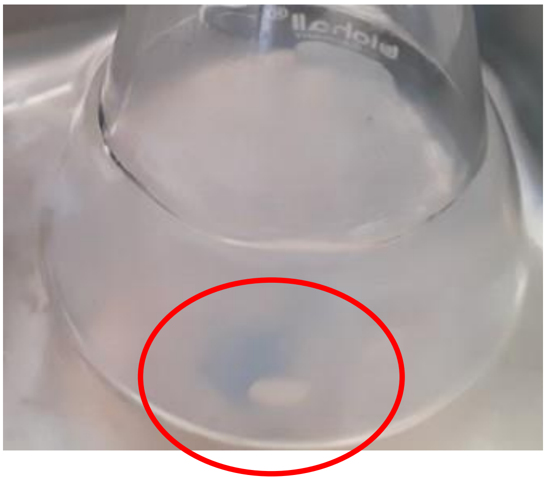

Figure S2. Agglomerated $BaTiO_3$, circled to highlight, after 90 minutes of ultrasonic treatment. The cloudy upper layer is a self-suspended $BaTiO_3$ suspension.

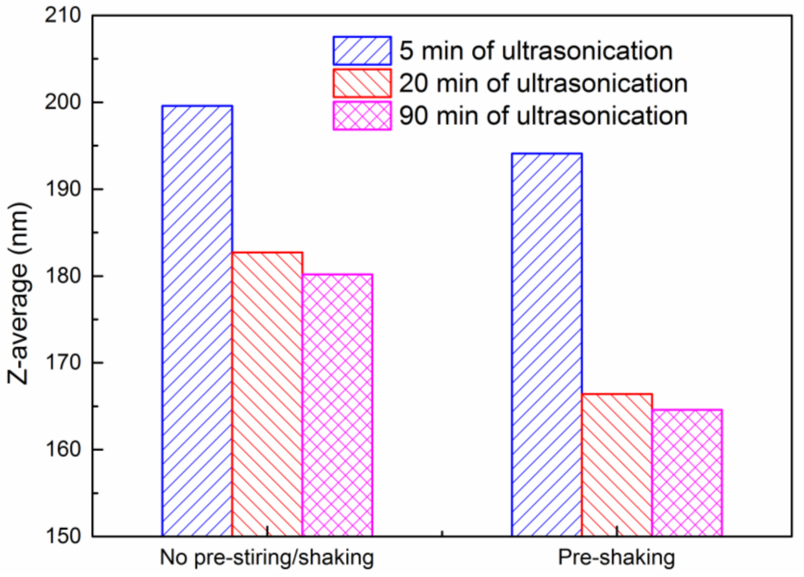

Figure S3. Z-average particle size obtained after different periods of ultrasonication. The x-axis indicates the pre-treatment conditions (BTO dosage: 100 mg·L^{-1}, solution: 10% MeOH, distance h: 10 mm).

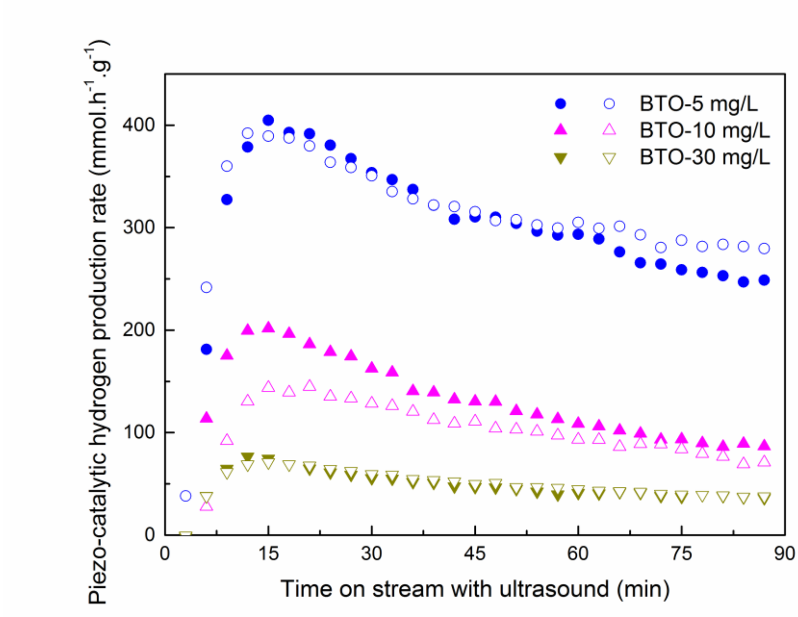

Figure S4. Reproducibility tests using different BTO dosages (solution: 10% MeOH, distance h: 13 mm). The same symbol shapes indicate the same experimental conditions.

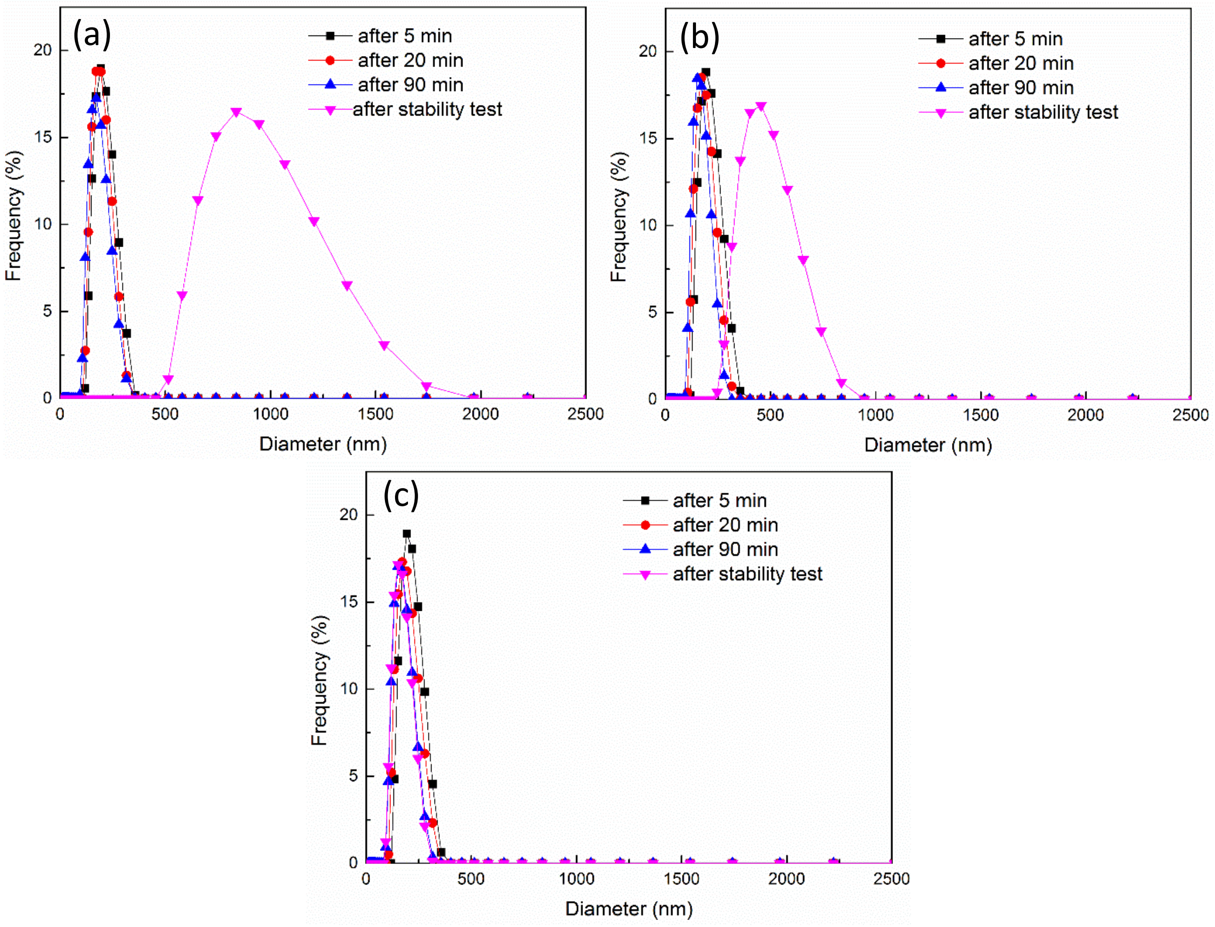

Figure S5. Size distribution of $BaTiO_3$ at a dosage of (a)100 $mg{\cdot}L^{-1}$, (b)30 $mg{\cdot}L^{-1}$ and (c) 5 $mg{\cdot}L^{-1}$ after 5, 20, 90 min of reaction and after stability tests using DLS.

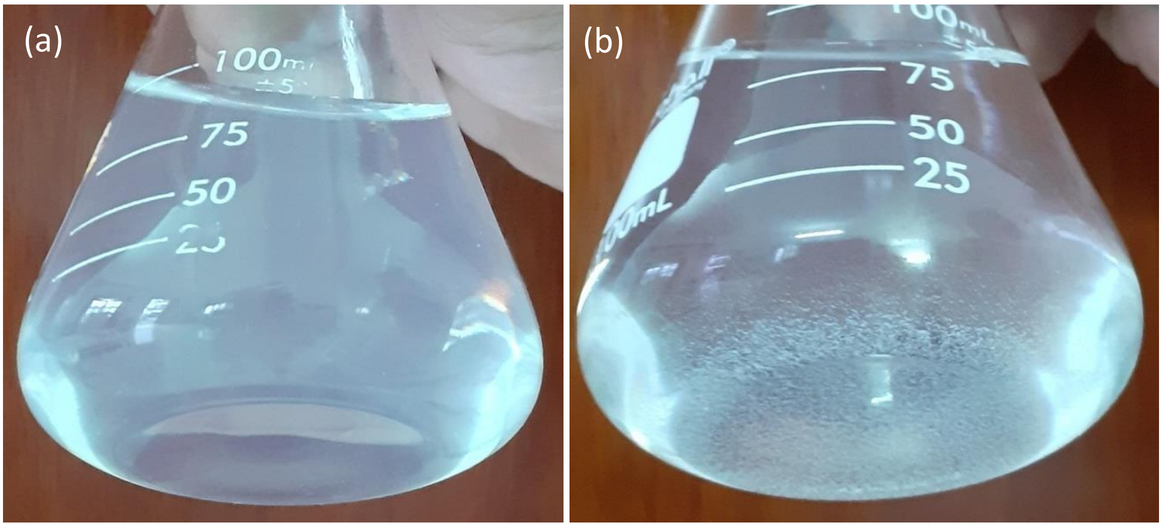

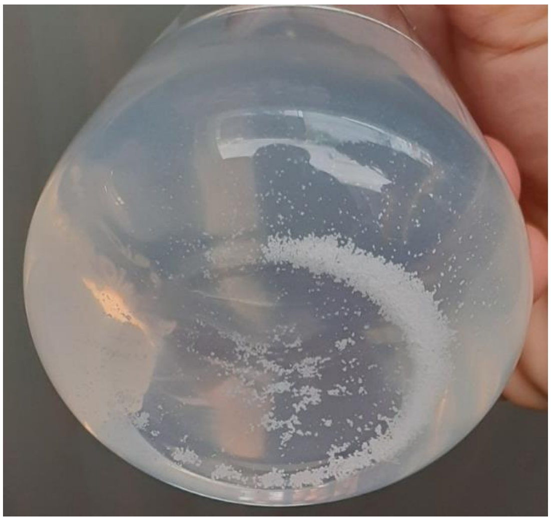

Figure S6. Photos of the reaction flasks with $BaTiO_3$ added at a dosage of (a) 5 $mg \cdot L^{-1}$ (b) 30 $mg \cdot L^{-1}$ and (c) 100 $mg \cdot L^{-1}$ after 240 min of ultrasonication and left overnight.

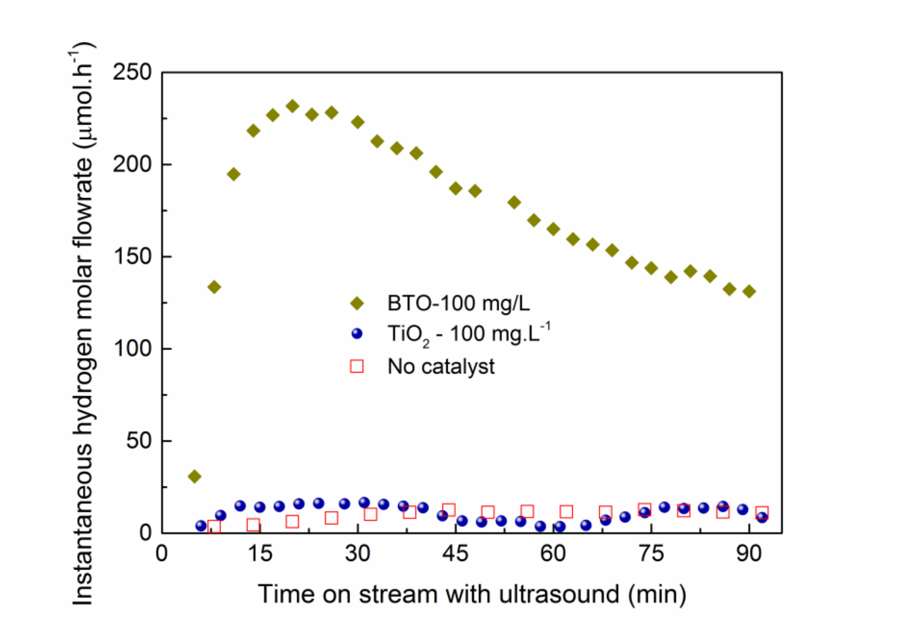

Figure S7. Hydrogen molar flowrate obtained from using $BaTiO_3$, a non-ferroelectric control (in the presence ofTiO_2 power) and a blank control (in the absence of any particles) in a 10% MeOH water solution.
